# Efficacy and Safety of Antifibrinolytic Agents in Reducing Perioperative Blood Loss and Transfusion Requirements in Scoliosis Surgery: A Systematic Review and Meta-Analysis

**DOI:** 10.1371/journal.pone.0137886

**Published:** 2015-09-18

**Authors:** Meng Wang, Xin-Feng Zheng, Lei-Sheng Jiang

**Affiliations:** Department of Orthopaedic Surgery, Xinhua Hospital, Shanghai Jiaotong University School of Medicine, Shanghai 200092, China; University of Toronto, CANADA

## Abstract

**Background:**

Routine use of antifibrinolytic agents in spine surgery is still an issue of debate.

**Objective:**

To gather scientific evidence for the efficacy and safety of antifibrinolytic agents including aprotinin, tranexamic acid (TXA) and epsilon aminocaproic acid (EACA, traditionally known as Amicar) in reducing perioperative blood loss and transfusion requirements in scoliosis surgery.

**Methods:**

We conducted a systematic review and meta-analysis for randomized controlled trials (RCTs), retrospective case-control studies, and retrospective cohort studies on the use of antifibrinolytic agents in scoliosis surgery by searching in the MEDLINE and EMBASE databases and the Cochrane Database of Systematic Reviews and Controlled Trials of papers published from January 1980 through July 2014. Safety of the antifibrinolytic agents was evaluated in all included studies, while efficacy was evaluated in RCTs.

**Results:**

Eighteen papers with a total of 1,158 patients were eligible for inclusion in this study. Among them, 8 RCTs with 450 patients were included for evaluation of pharmacologic efficacy (1 RCT was excluded because of a lack of standard deviation data). Mean blood loss was reduced in patients with perioperative use of antifibrinolytic agents by 409.25 ml intraoperatively (95% confidence interval [CI], 196.57–621.94 ml), 250.30 ml postoperatively (95% CI, 35.31–465.30), and 601.40 ml overall (95% CI, 306.64–896.16 ml). The mean volume of blood transfusion was reduced by 474.98 ml (95% CI, 195.30–754.67 ml). The transfusion rate was 44.6% (108/242) in the patients with antifibrinolytic agents and 68.3% (142/208) in the patients with placebo. (OR 0.38; 95% CI; 0.25–0.58; *P*<0.00001, I^2^ = 9%). All studies were included for evaluation of safety, with a total of 8 adverse events reported overall (4 in the experimental group and 4 in the control group).

**Conclusion:**

The systematic review and meta-analysis indicated that aprotinin, TXA, and EACA all significantly reduced perioperative blood loss and transfusion requirements in scoliosis surgery. There was no evidence that the use of antifibrinolytic agents was a risk factor for adverse events, especially thromboembolism, in scoliosis surgery.

## Introduction

Scoliosis, which is defined as a lateral curvature of the spine, is the most common deformity of the spine and has been focused for centuries by physicians[1]. Multi-segmental spinal instrumentation and fusion, and sometimes osteotomy were effective methods of correcting scoliosis, but were commonly associated with massive blood loss requiring blood transfusion[2–7]. Either allogenic or autologous blood transfusions can increase the length of hospital stay and medical cost. Allogenic blood transfusion in particular increased the rate of infectious disease, hemolytic reaction, septicemia, acute lung injury, severe immunoreaction, coagulation disorders, renal injury or failure, and even death[8–12].

Since the 1990s, antifibrinolytic agents, such as aprotinin, tranexamic acid (TXA) and epsilon aminocaproic acid (EACA), have been suggested to use in complex surgery to reduce bleeding. Aprotinin has a role of antiplasmin by inhibiting serine protease while TXA and EACA bind with the receptor competing to lysine to suppress fibrinolysis. More and more evidence revealed that these antifibrinolytic agents were successful to reduce perioperative blood loss and blood transfusion requirements in major pediatric surgery, cardiac surgery, total hip replacement arthroplasty and total knee replacement arthroplasty[13–15]. However, the efficacy and safety of these agents in scoliosis surgery remains yet to be determined.

The objective of this systematic review and meta-analysis was to determine whether the antifibrinolytic agents including aprotinin, TXA and EACA could effectively and safely reduce perioperative blood loss and transfusion requirements in scoliosis surgery. We followed the PRISMA guidelines to help improve reporting quality of our study (see S1 PRISMA Checklist).

## Materials and Methods

### Data Sources and Search Strategy

Interrelated randomized controlled trials (RCTs) and retrospective studies were distinguished in the MEDLINE and EMBASE databases and the Cochrane Database of Systematic Reviews and Controlled Trials from January 1980 through July 2014. Key words used in the search included antifibrinolytic agents, tranexamic acid, epsilon aminocaproic acid, aprotinin, spinal curvatures, scoliosis, kyphosis, lordosis, posterior lumbar spine fusion, randomized controlled trial, and comparative study. Once studies met the eligible criteria, they would be included even published in gray literature. The search was carried out without any linguistic restriction. Two investigators independently reviewed the title, abstract, and the full text of all articles. Eligible trials were chosen according to the inclusion criteria.

### Study Eligibility Criteria and Exclusion Criteria

Articles were selected on the basis of the following criteria: subjects were diagnosed with scoliosis and received correction surgery. Articles were excluded if the patients had one of the following conditions: (1) severe cardiopulmonary disease, hepatic or renal dysfunction; (2) extension of prothrombin time (PT) and activated partial thromboplastin time (APTT), decrease of platelet counts; (3) medical history of coagulation disorders; (4) intake of any anticoagulant drug within a week before surgery.

### Assessment of Risk of Bias

According to the Cochrane guidelines[16], two independent researchers and a managing reviewer assessed the included studies for risk of bias. Cochrane bias scale is composed of 7 parts: random sequence generation, allocation concealment, blinding of participants and personnel, blinding of outcome data, incomplete outcome data, selective reporting and other bias. Based on the report and appropriateness of methods, the included studies were graded accordingly: (1) low risk (methods were indicated and proper); (2) high risk (methods were indicated but improper); or (3) moderate risk (methods were not indicated).

### Data Collection

Two reviewers specialized in scoliosis extracted relevant data independently. If there was any disagreement, it would be decided by the senior professional reviewer. The data extracted was mainly divided into two parts, study characteristics and measuring outcomes. Study characteristics included the following items: the name of first author, year of publication, sample size, intervention, dose, number of levels fused, transfusion trigger and surgical procedure. Total blood loss, intraoperative blood loss, postoperative blood loss, blood transfusion volume and transfusion rate were extracted as the measuring outcomes of effectiveness while adverse event (thromboembolism, cerebrovascular accident, seizure, myocardial infarction, renal dysfunction or failure and even death) were extracted as the measuring outcomes of safety.

### Data Analysis

The data extracted was processed by Review Manager 5.0 software with a random-effect model. The odds ratio (OR) and 95% confidence interval (CI) were as statistical data to process dichotomous variables. If measuring outcome in the studies maintained uniformly standard, mean difference and 95% CI were as statistical data to process continuous variables. Otherwise, the standardized mean difference and 95% CI were calculated. The size of heterogeneity was calculated by the χ^2^ statistic, including *P* value and I^2^. If *P* > .10 and I^2^ ≤ 50%, the fixed-effect model would be applied on the meta-analysis. The random-effect model was used if *P* ≤ .10, I^2^ > 50%. The included studies were also subgrouped according to the specific pharmacologic interventions (aprotinin, or TXA, or EACA).

## Results

### Description of Study

**Fig 1:**
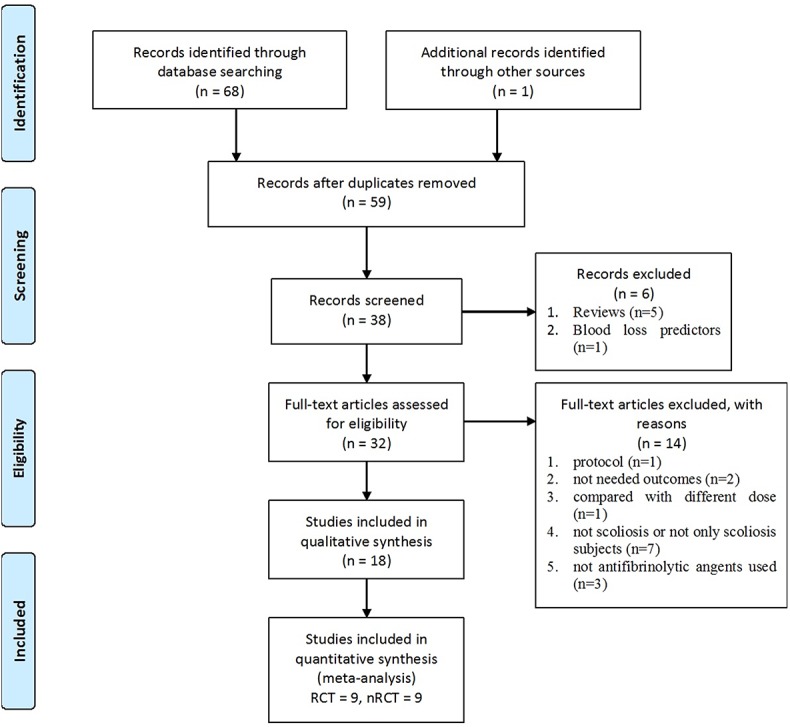
Flow diagram for study selection process in this meta-analysis.

The search identified 59 articles (Fig 1), of which 27 were excluded. Excluded studies included case reports, reviews, critical articles, and articles that did not match the inclusion criteria. Of the remaining 32 articles, 12 were RCTs and 20 were retrospective studies. After a full text reading, 3 randomized controlled trials and 11 retrospective studies were further excluded because of incompatible protocol specifications, use of outcome measures that were not specified in the meta-analysis, inclusion of patients without scoliosis, no use of antifibrinolytic agents, or comparisons involving different doses of antifibrinolytic agent.

In total, 18 studies with 1,158 patients fitted the criteria and were included in the meta-analysis. Among them, there were 9 RCTs[17–25], 7 retrospective case-control studies[26–32], and 2 retrospective cohort studies[33,34]. One[17] of the 9 remaining RCTs lacked required data (standard deviation of blood loss and blood transfusion volume), thus 8 RCTs with 450 patients were included for evaluation of pharmacologic efficacy. All the 18 studies were included for evaluation of pharmacologic safety. Among these 18 studies, five reported the use of TXA, 4 reported EACA, 6 reported aprotinin, 1 reported both TXA and EACA, and 2 reported both TXA and aprotinin (Table 1).

**Table 1:** Characteristics of the Studies Included.

Author	Year	Age(E/C)	Intervention	Size (E/C)	Dose(loading/ continuous infusion)	Number of levels fused (E/C)	Transfusion trigger	Type	Procedure	Design
Khoshhal	2003	14.5/14.1	aprotinin	43(15/28)	4mg/kg+1mg/kg/hr	10/10	Hgb < 7g/dL or Hct < 20%	IS	SF	RCT
Karapurkar	2002	12–25	aprotinin	49(24/25)	2*10^4 IU/kg+5*10^3 IU/kg/4hr	/	BEBL < 90% or Hct < 30% or Hgb < 10g/dl	KS	SF	RCT
Lentschener	1999	46±9/51±11	aprotinin	72(35/37)	2*10^6 KIU/kg+5*10^5 KIU/kg/hr	/	Hct < 26%	AD	SF	RCT
Cole	2003	13±4.7/12.2±3.8	aprotinin	44(21/23)	240mg/m2+56mg/m2/hr	14.5/12.2	Hgb < 8.5 g/dl or Hct < 27%	NS	SF	RCT
Tayyab	2008	47±15/41±15	aprotinin	82(41/41)	2*10^6KIU+5*10^5 KIU/hr	11.2/10.7	Hgb < 7g/dL	PS and SS	SF	nRCT
Kasimian	2008	12.4/14.8	aprotinin	31(14/17)	2*10^5KIU/kg+5*10^4 KIU/kg/hr	14.4/15.3	Hct≤24%	NS	SF	nRCT
Xu	2012	19.1±3.2/20.4±3.1	TXA	40(20/20)	20mg/kg+10mg/kg/hr	12.7/13.1	Hgb < 8g/dL	AIS	SF	RCT
Sethna	2005	13.6±1.8/14.0±2.0	TXA	44(23/21)	100mg/kg+10mg/kg/hr	14/13	Hct < 25%	PS and SS	SF	RCT
Neilipovitz	2001	14.1±2.1/13.7±2.5	TXA	40(22/18)	10mg/kg+1mg/kg/hr	15 /14	Hgb < 7g/dL	PS and SS	SF	RCT
Yagi	2012	15.2±2.9/15.5±3.0	TXA	106(43/63)	1g+100mg/hr	12.2/12.1	Hgb < 7g/dL	AIS	SF	nRCT
Lykissas	2013	14.7(12–19)/13.5(11–16)	TXA	49(25/24)	100mg+10mg/hr	10.7/12.6	Hgb < 7g/dL	AIS	SF	nRCT
Pineda	2004	13.5±1.6/14.5±1.3	EACA	36(19/17)	100mg/kg+10mg/kg/hr	12/12.1	Hgb < 7g/dL	IS	SF	RCT
Thompson	2008	14.0±2.4/12.3±2.8	EACA	96(62/34)	100mg/kg+10mg/kg/hr	15/14	Hgb < 7g/dL	NS	SF	nRCT
Pineda	2001	14.5±1.6/13.3±1.3	EACA	59(28/31)	100mg/kg+10mg/kg/hr	12/12	Hgb < 7g/dL	IS	SF	nRCT
Lorio	2013	N/A	EACA	33(17/16)	100mg/kg+10mg/kg/hr	/	Hgb < 7g/dL	AIS	SF with PO	nRCT
Verma	2014	15.3±2.4,14.6±1.9/15.0±2.4	TXAEACA	125(36,42/47)	TXA:10mg/kg +1mg/kg/hrEACA:100mg/kg +10mg/kg/hr	8.8,9.5/9.0	I: Hct < 25% P: Hct < 22%	AIS	SA	RCT
Newton	2012	13.7	aprotininTXA	136(30,42/64)	/	/	/	PS and SS	SF with VCR	nRCT
Khurana★	2012	26.3	aprotininTXA	73(28,26/17)	Aprotinin:1*10^6 KIU +5*10^5 KIU/hrTXA:2g+N/A	12.3,11.9/7.1	/	AD	SF	nRCT

E/C, experimental group/control group; TXA, tranexamic acid; EACA, epsilon aminocaproic acid; I, intraoperative; P, postoperative; Hct, hematocrit; PS, primary scoliosis; SS, secondary scoliosis; AD, adult deformity; NS, neuromuscular scoliosis; AIS, adolescent idiopathic scoliosis; IS, idiopathic scoliosis; KS, Kypho scoliosis; SF, spinal fusion; VCR, vertebral column resection; PO, Ponte osteotomy; BEBL, baseline estimated blood volume; RCT, randomized control trial; nRCT, non-randomized control trial.

★the operative approach in this study was either anterior, or posterior, or combined. In the others, all posterior

### Risk of Bias

**Fig 2:**
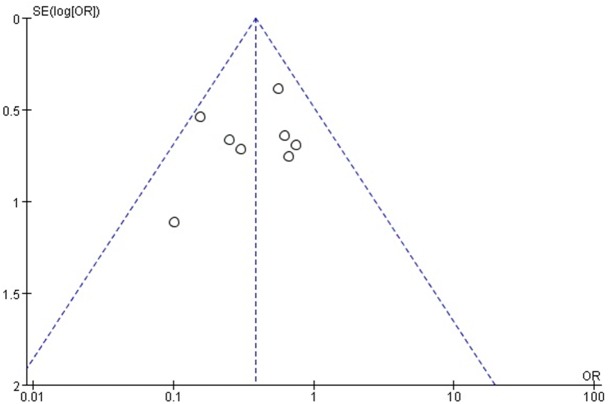
Funnel plot to assess publication bias.

The methodological quality of the included studies was assessed independently by two reviewers using Review Manager Software. The outcome was summarized in Table 2. Blood transfusion rates were used to generate a funnel plot analysis of publication bias (Fig 2). The asymmetric nature of the resultant plot indicated the presence of publication bias.

**Table 2:** Assessment of Risk of Bias in the Studies Included.

	Random sequence generation	Allocation concealment	Blinding of participants and personnel	Blinding of outcome data	Incomplete outcome data	Selective reporting	Other bias
RCT							
Lentschener 1999	L	L	L	L	L	L	L
Neilipovitz 2001	L	M	L	L	L	L	L
Karapurkar 2002	M	M	M	M	L	L	L
Cole 2003	L	L	L	L	L	L	L
Pineda 2004	M	L	L	L	L	L	L
Sethna 2005	L	L	L	L	L	L	L
Xu 2012	M	M	M	M	L	L	M
Verma 2014	L	L	L	L	L	L	L
Khoshhal 2003	M	M	M	M	L	L	L
nRCT							
Tayyab 2008	H	H	H	M	M	L	M
Newton 2012	H	H	H	M	M	L	M
Khurana 2012	H	H	H	M	M	L	L
Kasimian 2008	H	H	H	M	M	L	M
Yagi 2012	H	H	H	M	M	L	M
Thompson 2008	H	H	H	H	M	L	M
Lykissas 2013	H	H	H	H	M	L	M
Pineda 2001	H	H	H	M	M	L	M
Lorio 2013	H	H	H	M	M	L	M

L: low risk (methods were indicated and proper); H: high risk (methods were indicated but improper); M: moderate risk (methods were not indicated); RCT: randomized control trial; nRCT: non-randomized control trial.

### Baseline Characteristics

The essential characteristics of the patients with antifibrinolytic agent (experimental group) were brought into correspondence with those with placebo (control group) in every included study. There were no significant differences in the baseline values of preoperative Cobb angle, scoliosis etiology classification, preoperative hemoglobin level, number of levels fused, surgical duration, and estimated blood volume or lifestyle factors between the two groups. The consistency of preoperative factors ensured to wipe off potential bias.

### Perioperative Blood Loss and Blood Transfusion Volume

**Fig 3:**
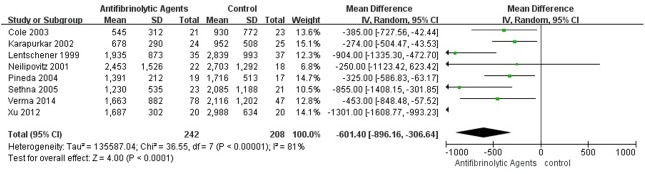
Forest plot diagram showing the effect of antifibrinolytic agents on total blood loss.

**Fig 4:**
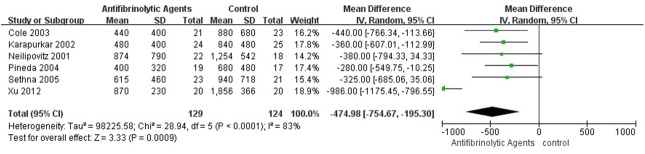
Forest plot diagram showing the effect of antifibrinolytic agents on blood transfusion requirements.

**Fig 5:**
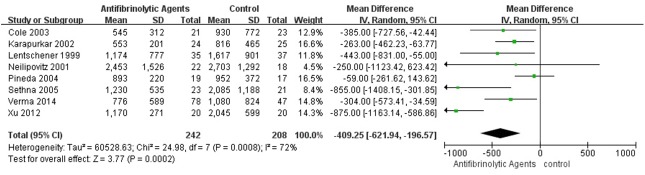
Forest plot diagram showing the effect of antifibrinolytic agents on intra-operative blood loss.

**Fig 6:**
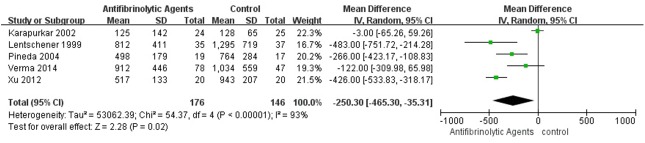
Forest plot diagram showing the effect of antifibrinolytic agents on post-operative blood loss.

High-quality evidence was available from 8 RCT studies (450 patients) in which antifibrinolytic agents were more effective than placebo in reducing total blood loss (weighted mean difference [WMD] = −601.40, 95% CI [−896.16, −306.64]; P < 0.0001; Fig 3). There was high-quality evidence from 6 studies (253 patients) in which antifibrinolytic agents were more effective than placebo in reducing perioperative blood transfusion volume (WMD = −474.98, 95% CI [−754.67, 195.30]; P = 0.0009; Fig 4). There was high-quality evidence from 8 studies (450 patients) in which antifibrinolytic agents were more effective than placebo in reducing intraoperative blood loss (WMD = −409.25, 95% CI [−621.94, −196.57], P = 0.0002, Fig 5). There was high-quality evidence from 8 studies (450 patients) in which antifibrinolytic agents were more effective than placebo in reducing postoperative blood loss (WMD = −250.30, 95% CI [−465.30, −35.31), P = 0.02, Fig 6).

### Perioperative Blood Transfusion Rate

**Fig 7:**
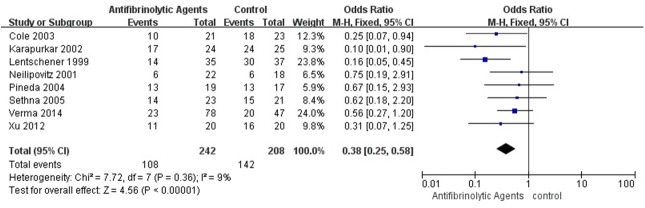
Forest plot diagram showing the effect of antifibrinolytic agents on blood transfusion rate.

The transfusion rate was 44.6% (108/242) in the patients with antifibrinolytic agents and 68.3% (142/208) in the patients with placebo. There was high-quality evidence from 8 studies (450 patients) in which antifibrinolytic agents were more effective than placebo in reducing perioperative blood transfusion rate (OR = 0.38, 95% CI [0.25, 0.58], P < 0.00001, Fig 7).

### Adverse Event

**Fig 8:**
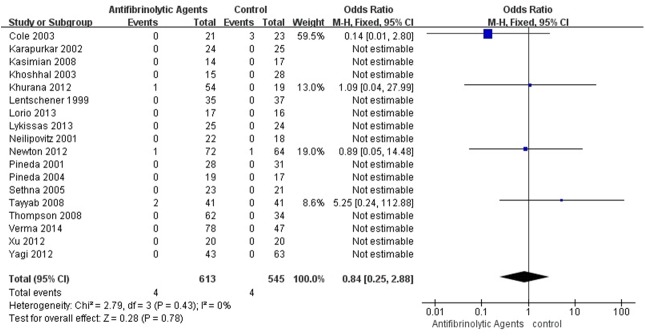
Forest plot diagram showing the effect of antifibrinolytic agents on adverse events rate.

Only 8 adverse events were reported in all the included studies. One pulmonary embolism and 3 deep vein thrombosis happened in the patients with antifibrinolytic agents while other 4 deep vein thrombosis occurred in the patients with placebo. There was moderate-quality evidence from the 18 studies (1,158 patients) in which no significant difference was found between antifibrinolytic agents and placebo in the rate of adverse events (OR = 0.84, 95% CI [0.25, 2.88], P = 0.78; Fig 8).

### Subgroup Analysis

The studies were divided into three subgroups according to the use of specific antifibrinolytic agent (Table 3). TXA, EACA and aprotinin were all more effective than placebo in reducing perioperative blood loss and blood transfusion requirements. Both TXA and aprotinin were more effective than placebo in reducing intraoperative blood loss and blood transfusion rate. No significant difference existed between EACA and placebo, or between aprotinin and placebo in reducing postoperative blood loss. One pulmonary embolism occurred in TXA group and 3 deep vein thromboses in aprotinin group. No significant difference existed between TXA and placebo, or between aprotinin and placebo in the adverse event rate. No adverse event was reported in the EACA group.

**Table 3:** Different Antifibrinolytic Agents Subgroup Analysis.

Outcome and subgroup	Studies	Participants	Statistical method	Effect estimate	p	X^2^	I^2^
Intraoperative blood loss							
TXA	4	207	Mean difference (IV, random, 95% CI)	−605.12, (−974.02, −236.22)	0.001	8.49	65%
Amicar	2	125	Mean difference (IV, random, 95% CI)	−162.83, (−405.94, 80.27)	0.19	1.95	49%
Aprotinin	3	165	Mean difference (IV, random, 95% CI)	−318.39, (−475.80, −160.98)	<.0001	0.84	0%
Postoperative blood loss							
TXA	2	123	Mean difference (IV, random, 95% CI)	−360.97, (−531.18, −190.77)	<.0001	2.14	53%
Amicar	2	125	Mean difference (IV, random, 95% CI)	−150.99, (−393.38, 91.41)	0.22	3.55	72%
Aprotinin	2	121	Mean difference (IV, random, 95% CI)	−224.47, (−693.46, 244.52)	0.35	11.63	91%
Total blood loss							
TXA	4	207	Mean difference (IV, random, 95% CI)	−828.60, (−1285.48, −371.72)	0.0004	9.96	70%
Amicar	2	125	Mean difference (IV, random, 95% CI)	−329.34, (−552.89, −105.78)	0.004	0	0%
Aprotinin	3	165	Mean difference (IV, random, 95% CI)	−482.47, (−821.28, −143.66)	0.005	6.4	69%
Perioperative Blood Transfusion Requirements							
TXA	3	124	Mean difference (IV, random, 95% CI)	−587.00, (−1073.03, −100.96)	0.02	14.26	86%
Amicar	1	36	Mean difference (IV, random, 95% CI)	−280.00, (−549.75, −10.25)	0.04	N/A	N/A
Aprotinin	2	93	Mean difference (IV, random, 95% CI)	−389.14, (−586.09, −192.19)	0.0001	0.15	0%
Transfusion rate							
TXA	4	207	Odds ratio (M-H, fixed, 95% CI)	0.53, (0.29, 0.95)	0.03	0.9	0%
Amicar	2	125	Odds ratio (M-H, fixed, 95% CI)	0.62, (0.29, 1.32)	0.21	0.01	0%
Aprotinin	3	165	Odds ratio (M-H, fixed, 95% CI)	0.17, (0.08, 0.37)	<.00001	0.59	0%
Adverse Event							
TXA	8	513	Odds ratio (M-H, fixed, 95% CI)	1.54, (0.09, 25.26)	0.76	N/A	N/A
Amicar	5	313	Odds ratio (M-H, fixed, 95% CI)	Not estimable			
Aprotinin	8	469	Odds ratio (M-H, fixed, 95% CI)	0.96, (0.28, 3.29)	0.95	3.26	8%

## Discussion

Effective control of perioperative blood loss helps increase the patient’s tolerance to surgery, decrease postoperative infection rate, reduce the probability of blood transfusion, shorten hospital stays and decrease medical cost. This meta-analysis indicated that the antifibrinolytic agents aprotinin, TXA and EACA could reduce perioperative blood loss and blood transfusion volume effectively in scoliosis surgery. There was no evidence that use of these agents was a risk factor to increase the incidence of adverse events. TXA and aprotinin appeared more effective than EACA according to the results of this analysis.

Fibrinolysis is the liquefied process of fibrin formed in blood clotting. It consists of two elementary steps, activation of plasminogen and degradation of fibrin[35–37]. Aprotinin was first extracted from bovine tissues and is now also produced by recombinant technology as a serine proteinase inhibitor[38,39]. The drug was a pioneering antifibrinolytic agent used in surgery. It was first used in the 1960s to reduce blood loss in major cardiac surgery involving cardiopulmonary bypass[17]. In 1993, aprotinin was approved by the Food and Drug Administration (FDA) as a hemostatic and was widely used in coronary artery bypass graft procedures. However, in 2006, Mangano et al[40] performed an observational study involving 4,374 patients undergoing revascularization which investigated adverse events associated with aprotinin in cardiac surgery. This study indicated that the use of aprotinin might have a positive correlation with renal failure, myocardial infarction, cerebrovascular accident, and death[40,41]. Just because of this, aprotinin was withdrawn by FDA in 2007 due to safety concerns. It was not likely approved for use again in the United States or Canada in cardiac surgery. More recently, lysine analogs (TXA and EACA) have replaced aprotinin in reducing perioperative blood loss and blood transfusion requirements due to the improved safety profile of these agents. Both EACA and TXA inhibited plasminogen from binding to the surface of fibrin[42–45].

Few RCTs or observational studies have been reported directly comparing TXA, EACA, and aprotinin in terms of benefit in reducing perioperative blood loss and transfusion requirements. Makhija et al[44] and Verma et al[24] concluded that no significant difference was found between TXA and EACA in efficacy based on their studies of thoracic aortic surgery and scoliosis surgery, respectively. Compared with lysine analogs, aprotinin was more efficient in reducing perioperative blood loss [45], although there was controversy over whether it would also be able to decrease perioperative blood transfusion requirements more efficiently[45–47]. Our results were not completely consistent with these findings. This meta-analysis demonstrated that TXA and aprotinin were more effective in reducing perioperative blood loss and transfusion requirements in scoliosis surgery compared with EACA. However, this conclusion needs to be further confirmed because of the lack of well-designed RCTs directly comparing these agents.

The medical community has expressed doubts over the safety of antifibrinolytic agents over the past few years. Some large meta-analyses have shown that use of antifibrinolytic agents might increase the risk of death, MI, cerebrovascular accidents, seizure, and renal injury or failure[48–51]. In this analysis, a total of 18 papers including 1,158 patients were eligible for inclusion for evaluation of safety. Surprisingly, none of the included paper reported the above-mentioned adverse events in any patient. One possible reason might be the underlying disease that was operated. Stroke, MI, renal injury or failure and even death were the common complications of cardiac surgery so the rates of these adverse events would be high no matter whether antifibrinolytic agents were used or not. The other one might be due to our exclusion criteria in data collection that we excluded the patients who originally had severe cardiopulmonary disease, hepatic or renal dysfunction.

Use of antifibrinolytic agents is a theoretical risk factor for thromboembolism after operation. However, only 8 thromboembolic events were reported in our meta-analysis. Four happened in the patients with antifibrinolytic agents and the other 4 occurred in those with placebo. In the patients with antifibrinolytic agents, 1 pulmonary embolism (PE) happened in a patient with TXA, 3 deep vein thromboses (DVT) occurred in those with aprotinin, while none in the patients with EACA. Unlike extremity operations with high complications of DVT or PE, the underlying disease of spine that was operated in our meta-analysis had a very low rate of thromboembolism no matter whether antifibrinolytic agents were used or not. The low incidence of thromboembolic complications in our meta-analysis was largely consistent with previous papers. Sansur et al[52] reported that the rate of PE was 0.2% (12 cases), and the rate of DVT was 0.18% (9 cases) in a total of 4,980 operated cases of idiopathic adult scoliosis. Similarly, Jain et al[53] found that the incidence of venous thromboembolism in 21,955 children with spinal fusions was 0.21% (0.096–0.385%), while the rate of PE was 0.02% (0–0.06%). Thus, we believed that use of antifibrinolytic agents was not a risk factor for adverse events, particularly thromboembolism, in scoliosis surgery.

This study had some limitations. According to our search results and inclusion criteria, 9 RCTs and 9 observational studies were included. The small quantity of RCTs and incomplete data might reduce the quality of evidence and the strength of analysis. There was no clear attempt at random sequence generation, allocation concealment, blinding of participants and personnel, or blinding of outcome data in 2 RCTs[20,25] for efficacy evaluation. In addition, the age of the subjects in each study included was different; some studies were restricted to children, others involved adults. The pathogenesis of scoliosis in different studies varied; some were idiopathic and others were secondary in nature. Some studies proved that perioperative blood loss in secondary scoliosis surgery was much greater than that in idiopathic scoliosis surgery. In addition, there might be some performance bias due to surgical time, fusion levels, transfusion trigger, dose of antifibrinolytic agents and intraoperative MAP. Finally, there were 2 RCTs[24,25] presented in diagrammatic form (represented by the error bars which were measures directly), from which we extracted primary data by ratio-metric conversion, another potential source of bias.

## Conclusion

Aprotinin, TXA, and EACA were all able to reduce perioperative blood loss and transfusion requirements in scoliosis surgery. There was no evidence that use of antifibrinolytic agents was a risk factor for adverse events, especially thromboembolism, in scoliosis surgery. Nevertheless, the number of either total patients or adverse events was relatively small. Further multicenter, large-sample, double-blind RCTs are required to confirm the efficacy and safety of the three antifibrinolytic agents in spine surgery.

## Supporting Information

S1 PRISMA Checklist (DOC)

## References

[1] Asher MA, Burton DC. Adolescent idiopathic scoliosis: natural history and long term treatment effects. Scoliosis. 2006;1(1):2 10.1186/1748-7161-1-2 . 16759428 PMC1475645

[2] Luque ER. Segmental spinal instrumentation for correction of scoliosis. Clinical orthopaedics and related research. 1982;(163):192–8. . 7067252

[3] Storer SK, Vitale MG, Hyman JE, Lee FY, Choe JC, Roye DP Jr. Correction of adolescent idiopathic scoliosis using thoracic pedicle screw fixation versus hook constructs. Journal of pediatric orthopedics. 2005;25(4):415–9. . 15958886 10.1097/01.mph.0000165134.38120.87

[4] Bjerkreim I, Steen H, Brox JI. Idiopathic scoliosis treated with Cotrel-Dubousset instrumentation: evaluation 10 years after surgery. Spine. 2007;32(19):2103–10. 10.1097/BRS.0b013e318145a54a . 17762812

[5] Guay J, Reinberg C, Poitras B, David M, Mathews S, Lortie L, et al A trial of desmopressin to reduce blood loss in patients undergoing spinal fusion for idiopathic scoliosis. Anesthesia and analgesia. 1992;75(3):405–10. . 1510262 10.1213/00000539-199209000-00016

[6] Doi T, Harimaya K, Matsumoto Y, Taniguchi H, Iwamoto Y. Peri-operative blood loss and extent of fused vertebrae in surgery for adolescent idiopathic scoliosis. Fukuoka igaku zasshi = Hukuoka acta medica. 2011;102(1):8–13. . 21516986

[7] Koerner JD, Patel A, Zhao C, Schoenberg C, Mishra A, Vives MJ, et al Blood loss during posterior spinal fusion for adolescent idiopathic scoliosis. Spine. 2014;39(18):1479–87. 10.1097/BRS.0000000000000439 . 24859581

[8] Karkouti K, Dattilo KM. Perioperative hemostasis and thrombosis. Canadian journal of anaesthesia = Journal canadien d'anesthesie. 2006;53(12):1260–2. 10.1007/BF03021588 . 17142661

[9] Cardone D, Klein AA. Perioperative blood conservation. European journal of anaesthesiology. 2009;26(9):722–9. 10.1097/EJA.0b013e32832c5280 . 19448549

[10] Kumar A. Perioperative management of anemia: limits of blood transfusion and alternatives to it. Cleveland Clinic journal of medicine. 2009;76 Suppl 4:S112–8. 10.3949/ccjm.76.s4.18 . 19880828

[11] Soviero F, Geraci A, Termine S, Sanfilippo A, Maritano RM, D'Arienzo M, et al Bleeding in orthopaedic surgery: the role of blood transfusion and erythropoietin alpha. Acta bio-medica: Atenei Parmensis. 2010;81(2):125–9. . 21305877

[12] Varney SJ, Guest JF. The annual cost of blood transfusions in the UK. Transfusion medicine. 2003;13(4):205–18. . 12880391 10.1046/j.1365-3148.2003.00443.x

[13] Schouten ES, van de Pol AC, Schouten AN, Turner NM, Jansen NJ, Bollen CW. The effect of aprotinin, tranexamic acid, and aminocaproic acid on blood loss and use of blood products in major pediatric surgery: a meta-analysis. Pediatric critical care medicine: a journal of the Society of Critical Care Medicine and the World Federation of Pediatric Intensive and Critical Care Societies. 2009;10(2):182–90. 10.1097/PCC.0b013e3181956d61 . 19188875

[14] Brown JR, Birkmeyer NJ, O'Connor GT. Meta-analysis comparing the effectiveness and adverse outcomes of antifibrinolytic agents in cardiac surgery. Circulation. 2007;115(22):2801–13. 10.1161/CIRCULATIONAHA.106.671222 . 17533182

[15] Alshryda S, Sukeik M, Sarda P, Blenkinsopp J, Haddad FS, Mason JM. A systematic review and meta-analysis of the topical administration of tranexamic acid in total hip and knee replacement. The bone & joint journal. 2014;96-B(8):1005–15. 10.1302/0301-620X.96B8.33745 . 25086114

[16] J. H, D A. Assessing risk of bias in included studies Cochrane Handbook for Systematic Reviews of Interventions Version 5.0.0. The Cochrane Collaboration 2008;Available: http://hiv.cochrane.org/sites/hiv.cochrane.org/files/uploads/Ch08_Bias.pdf (Accessed 2008).

[17] Khoshhal K, Mukhtar I, Clark P, Jarvis J, Letts M, Splinter W. Efficacy of aprotinin in reducing blood loss in spinal fusion for idiopathic scoliosis. Journal of pediatric orthopedics. 2003;23(5):661–4. . 12960633 10.1097/00004694-200309000-00017

[18] Cole JW, Murray DJ, Snider RJ, Bassett GS, Bridwell KH, Lenke LG. Aprotinin reduces blood loss during spinal surgery in children. Spine. 2003;28(21):2482–5. 10.1097/01.BRS.0000090835.45437.7F . 14595168

[19] Florentino-Pineda I, Thompson GH, Poe-Kochert C, Huang RP, Haber LL, Blakemore LC. The effect of amicar on perioperative blood loss in idiopathic scoliosis: the results of a prospective, randomized double-blind study. Spine. 2004;29(3):233–8. . 14752343 10.1097/01.brs.0000109883.18015.b9

[20] Karapurkar A KA, Naik L. aprotinin, to reduced perioperative blood loss in scoliosis surgery Indian J Anaesth 2002;46(5):378–80.

[21] Lentschener C, Cottin P, Bouaziz H, Mercier FJ, Wolf M, Aljabi Y, et al Reduction of blood loss and transfusion requirement by aprotinin in posterior lumbar spine fusion. Anesthesia and analgesia. 1999;89(3):590–7. . 10475286 10.1097/00000539-199909000-00009

[22] Neilipovitz DT, Murto K, Hall L, Barrowman NJ, Splinter WM. A randomized trial of tranexamic acid to reduce blood transfusion for scoliosis surgery. Anesthesia and analgesia. 2001;93(1):82–7. . 11429344 10.1097/00000539-200107000-00018

[23] Sethna NF, Zurakowski D, Brustowicz RM, Bacsik J, Sullivan LJ, Shapiro F. Tranexamic acid reduces intraoperative blood loss in pediatric patients undergoing scoliosis surgery. Anesthesiology. 2005;102(4):727–32. . 15791100 10.1097/00000542-200504000-00006

[24] Verma K, Errico T, Diefenbach C, Hoelscher C, Peters A, Dryer J, et al The relative efficacy of antifibrinolytics in adolescent idiopathic scoliosis: a prospective randomized trial. The Journal of bone and joint surgery American volume. 2014;96(10):e80 10.2106/JBJS.L.00008 . 24875032

[25] Xu C, Wu A, Yue Y. Which is more effective in adolescent idiopathic scoliosis surgery: batroxobin, tranexamic acid or a combination? Archives of orthopaedic and trauma surgery. 2012;132(1):25–31. 10.1007/s00402-011-1390-6 . 21909815

[26] Kasimian S, Skaggs DL, Sankar WN, Farlo J, Goodarzi M, Tolo VT. Aprotinin in pediatric neuromuscular scoliosis surgery. European spine journal: official publication of the European Spine Society, the European Spinal Deformity Society, and the European Section of the Cervical Spine Research Society. 2008;17(12):1671–5. 10.1007/s00586-008-0790-y . 18820953 PMC2587686

[27] Khurana A, Guha A, Saxena N, Pugh S, Ahuja S. Comparison of aprotinin and tranexamic acid in adult scoliosis correction surgery. European spine journal: official publication of the European Spine Society, the European Spinal Deformity Society, and the European Section of the Cervical Spine Research Society. 2012;21(6):1121–6. 10.1007/s00586-012-2205-3 . 22402839 PMC3366133

[28] Newton PO, Bastrom TP, Emans JB, Shah SA, Shufflebarger HL, Sponseller PD, et al Antifibrinolytic agents reduce blood loss during pediatric vertebral column resection procedures. Spine. 2012;37(23):E1459–63. 10.1097/BRS.0b013e31826c9fe4 . 22872217

[29] Tayyab NA, Mariller MM, Rivlin M, Berekashvili K, Bitan FD, Casden AM, et al Efficacy of aprotinin as a blood conservation technique for adult deformity spinal surgery: a retrospective study. Spine. 2008;33(16):1775–81. 10.1097/BRS.0b013e31817b87c4 . 18628710

[30] Yagi M, Hasegawa J, Nagoshi N, Iizuka S, Kaneko S, Fukuda K, et al Does the intraoperative tranexamic acid decrease operative blood loss during posterior spinal fusion for treatment of adolescent idiopathic scoliosis? Spine. 2012;37(21):E1336–42. 10.1097/BRS.0b013e318266b6e5 . 22772572

[31] Thompson GH, Florentino-Pineda I, Poe-Kochert C, Armstrong DG, Son-Hing J. Role of Amicar in surgery for neuromuscular scoliosis. Spine. 2008;33(24):2623–9. 10.1097/BRS.0b013e318187c046 . 18981961

[32] Lykissas MG, Crawford AH, Chan G, Aronson LA, Al-Sayyad MJ. The effect of tranexamic acid in blood loss and transfusion volume in adolescent idiopathic scoliosis surgery: a single-surgeon experience. Journal of children's orthopaedics. 2013;7(3):245–9. 10.1007/s11832-013-0486-7 . 24432083 PMC3672458

[33] Florentino-Pineda I, Blakemore LC, Thompson GH, Poe-Kochert C, Adler P, Tripi P. The Effect of epsilon-aminocaproic acid on perioperative blood loss in patients with idiopathic scoliosis undergoing posterior spinal fusion: a preliminary prospective study. Spine. 2001;26(10):1147–51. . 11413428 10.1097/00007632-200105150-00011

[34] Iorio J, Bennett JT, Orlando G, Singla A, Dakwar E, Bonet H, et al Does Amicar affect blood loss in patients with adolescent idiopathic scoliosis treated with pedicle screws and Ponte osteotomies? Surgical technology international. 2013;23:291–5. . 23975447

[35] Ortmann E, Besser MW, Klein AA. Antifibrinolytic agents in current anaesthetic practice. British journal of anaesthesia. 2013;111 (4)(549–63 (2013)). 10.1093/bja/aet154 23661406

[36] K G, EGD T, JH M. Normal haemostasis In:Hoffbrand AV, Catovsky D, Tuddenham EGD, eds. 2011;Postgraduate Haematology(Oxford:Wiley-Blackwell, 2011).

[37] U H, J H, V M. Therapywith Antifibrinolytic Agents In: Colman RW, Hirsch J, Marder V, Clowes A, George J, eds. 2001;Hemostasis and Thrombosis: Basic Principles and Clinical Practice(Philadelphia: LippincottWilliams &Wilkins, 2001).

[38] Fritz H, Wunderer G. Biochemistry and applications of aprotinin, the kallikrein inhibitor from bovine organs. Arzneimittel-Forschung. 1983;33(4):479–94. . 6191764

[39] Davis R, Whittington R. Aprotinin. A review of its pharmacology and therapeutic efficacy in reducing blood loss associated with cardiac surgery. Drugs. 1995;49(6):954–83. . 7543841 10.2165/00003495-199549060-00008

[40] Mangano DT, Tudor IC, Dietzel C, Multicenter Study of Perioperative Ischemia Research G, Ischemia R, Education F. The risk associated with aprotinin in cardiac surgery. The New England journal of medicine. 2006;354(4):353–65. 10.1056/NEJMoa051379 . 16436767

[41] Mangano DT, Miao Y, Vuylsteke A, Tudor IC, Juneja R, Filipescu D, et al Mortality associated with aprotinin during 5 years following coronary artery bypass graft surgery. JAMA: the journal of the American Medical Association. 2007;297(5):471–9. 10.1001/jama.297.5.471 . 17284697

[42] Samama CM. A direct antifibrinolytic agent in major orthopedic surgery. Orthopedics. 2004;27(6 Suppl):s675–80. . 15239556 10.3928/0147-7447-20040602-09

[43] Hardy JF, Belisle S. Natural and synthetic antifibrinolytics in adult cardiac surgery: efficacy, effectiveness and efficiency. Canadian journal of anaesthesia = Journal canadien d'anesthesie. 1994;41(11):1104–12. 10.1007/BF03015662 . 7530172

[44] Makhija N, Sarupria A, Kumar Choudhary S, Das S, Lakshmy R, Kiran U. Comparison of epsilon aminocaproic acid and tranexamic Acid in thoracic aortic surgery: clinical efficacy and safety. Journal of cardiothoracic and vascular anesthesia. 2013;27(6):1201–7. 10.1053/j.jvca.2013.04.003 . 24050855

[45] Henry DA, Carless PA, Moxey AJ, O'Connell D, Stokes BJ, Fergusson DA, et al Anti-fibrinolytic use for minimising perioperative allogeneic blood transfusion. The Cochrane database of systematic reviews. 2011;(3):CD001886 10.1002/14651858.CD001886.pub4 . 21412876 PMC4234031

[46] Martin K, Gertler R, MacGuill M, Mayr NP, Hapfelmeier A, Horer J, et al Replacement of aprotinin by epsilon-aminocaproic acid in infants undergoing cardiac surgery: consequences for blood loss and outcome. British journal of anaesthesia. 2013;110(4):615–21. 10.1093/bja/aes430 . 23213034

[47] Scott JP, Costigan DJ, Hoffman GM, Simpson PM, Dasgupta M, Punzalan R, et al Increased recombinant activated factor VII use and need for surgical reexploration following a switch from aprotinin to epsilon-aminocaproic acid in infant cardiac surgery. Journal of clinical anesthesia. 2014;26(3):204–11. 10.1016/j.jclinane.2013.10.015 . 24809789

[48] Henry D, Carless P, Fergusson D, Laupacis A. The safety of aprotinin and lysine-derived antifibrinolytic drugs in cardiac surgery: a meta-analysis. CMAJ: Canadian Medical Association journal = journal de l'Association medicale canadienne. 2009;180(2):183–93. 10.1503/cmaj.081109 . 19050037 PMC2621296

[49] Hutton B, Joseph L, Fergusson D, Mazer CD, Shapiro S, Tinmouth A. Risks of harms using antifibrinolytics in cardiac surgery: systematic review and network meta-analysis of randomised and observational studies. Bmj. 2012;345:e5798 10.1136/bmj.e5798 . 22968722 PMC3438881

[50] Meybohm P, Herrmann E, Nierhoff J, Zacharowski K. Aprotinin may increase mortality in low and intermediate risk but not in high risk cardiac surgical patients compared to tranexamic acid and epsilon-aminocaproic acid—a meta-analysis of randomised and observational trials of over 30.000 patients. PloS one. 2013;8(3):e58009 10.1371/journal.pone.0058009 . 23483965 PMC3590293

[51] Martin K, Wiesner G, Breuer T, Lange R, Tassani P. The risks of aprotinin and tranexamic acid in cardiac surgery: a one-year follow-up of 1188 consecutive patients. Anesthesia and analgesia. 2008;107(6):1783–90. 10.1213/ane.0b013e318184bc20 . 19020118

[52] Sansur CA, Smith JS, Coe JD, Glassman SD, Berven SH, Polly DW Jr., et al Scoliosis research society morbidity and mortality of adult scoliosis surgery. Spine. 2011;36(9):E593–7. 10.1097/BRS.0b013e3182059bfd . 21325989

[53] Jain A, Karas DJ, Skolasky RL, Sponseller PD. Thromboembolic complications in children after spinal fusion surgery. Spine. 2014;39(16):1325–9. 10.1097/BRS.0000000000000402 . 25010014

